# Feasibility of Fit24, a Digital Diabetes Prevention Program for Hispanic Adolescents: Qualitative Evaluation Study

**DOI:** 10.2196/54595

**Published:** 2024-05-17

**Authors:** Erica G Soltero, Salma M Musaad, Teresia M O’Connor, Debbe Thompson, Keith Norris, Bettina M Beech

**Affiliations:** 1 USDA/ARS Children's Nutrition Research Center Department of Pediatrics Baylor College of Medicine Houston, TX United States; 2 Division of General Internal Medicine and Health Services Research David Geffen School of Medicine University of California Los Angeles, CA United States; 3 UH Population Health University of Houston Houston, TX United States

**Keywords:** health disparities, diabetes prevention, Mexican youth, physical activity, sleep, digital health

## Abstract

**Background:**

Digital health interventions are promising for reaching and engaging high-risk youth in disease prevention opportunities; however, few digital prevention interventions have been developed for Hispanic youth, limiting our knowledge of these strategies among this population.

**Objective:**

This study qualitatively assessed the feasibility and acceptability of Fit24, a 12-week goal-setting intervention that uses a Fitbit watch (Fitbit Inc) and theoretically grounded SMS text messages to promote physical activity and sleep among Hispanic adolescents (aged between 14 and 16 years) with obesity.

**Methods:**

After completing the intervention, a subsample of youth (N=15) participated in an in-depth interview. We categorized the themes into dimensions based on participant perspectives using the Practical, Robust Implementation, and Sustainability Model (PRISM) framework.

**Results:**

Participants shared positive perceptions of wearing the Fitbit and receiving SMS text messages. Youth were highly engaged in monitoring their behaviors and perceived increased activity and sleep. Almost all youth organically received social support from a peer or family member and suggested the use of a group chat or team challenge for integrating peers into future interventions. However, most youth also expressed the need to take personal responsibility for the change in their behavior. Barriers that impacted the feasibility of the study included the skin-irritating material on the Fitbit watch band and environmental barriers (eg, lack of resources and school schedules), that limited participation in activity suggestions. Additionally, sync issues with the Fitbit limited the transmission of data, leading to inaccurate feedback.

**Conclusions:**

Fit24 is a promising approach for engaging Hispanic youth in a diabetes prevention program. Strategies are needed to address technical issues with the Fitbit and environmental issues such as message timing. While integrating peer social support may be desired by some, peer support strategies should be mindful of youth’s desire to foster personal motivation for behavior change. Findings from this study will inform future diabetes prevention trials of Fit24 and other digital health interventions for high-risk pediatric populations.

## Introduction

Digital devices such as wearable personal activity trackers (eg, Fitbit watch [Fitbit Inc] and Apple Watch [Apple Inc]) have gained widespread popularity among consumers and can offer innovative solutions for promoting healthy behaviors like physical activity (PA) and sleep [1]. While most digital health interventions (DHIs) using wearable activity trackers have been conducted among adults, some have focused on children and adolescents. Youth-focused studies have demonstrated small to moderate improvements in daily total steps, moderate-to-vigorous PA, and total overall PA; however, others have reported no change in PA behaviors [2-4]. Youth-focused studies report the use of multiple behavior change techniques, integrate multiple theoretical frameworks, and often use activity trackers in combination with other digital devices and platforms, including mobile apps and SMS text messaging [1,5-8]. The heterogeneity in intervention strategies reported in the literature makes it difficult to determine the most effective approach for leveraging activity trackers as behavior change tools among youth [4].

Engaging youth as collaborators using co-design approaches can inform the development of DHIs, identifying the intervention strategies that are feasible and acceptable among youth [9]. Within co-design approaches, youth educate researchers on their needs, expectations, culture, and perspectives. This bidirectional transfer of knowledge guides the research from design to implementation and, ultimately, through broad dissemination [9-12]. Using qualitative and quantitative co-design methods, we developed Fit24, a 12-week goal setting Fitbit and SMS text messaging intervention in collaboration with a community sample of Hispanic adolescents with obesity [13]. Youth (n=20) completed in-depth interviews to identify barriers and facilitators of PA and sleep, desired behavior change techniques, preferences for support in line with self-determination theory (SDT), and desired goal-setting assistance and feedback. Information gleaned from these interviews was used to develop a bank of SMS text messages (n=125) that were reviewed and evaluated by research experts (n=6) and a subsample of Hispanic adolescents (n=5) that had previously completed an in-depth interview. Participants also provided contextual information to guide intervention strategies, such as the preferred frequency and timing of SMS text messaging, the impact of participation on family data plans, and family rules about texting and phone use.

The Fit24 intervention was implemented and assessed for feasibility among Hispanic adolescents (n=43, aged between 14 and 16 years) with obesity (BMI≥95th percentile) in a randomized, pilot intervention. The purpose of this study was to qualitatively assess the feasibility and acceptability of Fit24, the use of a Fitbit device, and theoretically grounded SMS text messages promoting PA and sleep. Participants included a subsample of Hispanic youth (N=15) with obesity who completed the intervention and participated in an exit interview. This study represents the next step in the co-design process by including youth as co-collaborators in the evaluation of this intervention. The valuable insights gained from this qualitative assessment will inform the implementation of DHIs among Hispanic youth and the next iteration of this intervention.

## Methods

### Participants

Youth were recruited from local pediatric clinics, community-based organizations (eg, Houston Public Library and Baker Ripley Community Developers), and by word of mouth. Interested participants were screened for eligibility using the following inclusion criteria: (1) self-identify as Hispanic; (2) between the ages of 14 and 16 years; (3) present with obesity, defined as BMI ≥95th percentile; and (4) have their own smartphone. Participants were excluded if they (1) took medication or were diagnosed with a condition that influences activity or sleep (eg, insomnia and sleep apnea), (2) experienced a recent hospitalization or injury that would impact activity or sleep, (3) were diagnosed with type 2 diabetes, (4) were pregnant, or (5) were enrolled in an exercise program or currently using a PA monitoring device.

### Fit24 Intervention

The intervention protocol has been previously published [13]. Participating youth were randomized (1:1 randomization) to the intervention or to a waitlist control group. The youth in the intervention received a Fitbit Charge 5 device and daily SMS text messages grounded in SDT [14]. Messages were designed to meet basic psychological needs by providing goal-setting assistance at the beginning of the week, tips, strategies, and suggestions to support goal attainment throughout the week, and feedback on goal attainment at the end of the week. Messages focused on PA for the first 3 weeks and focused on both PA and sleep starting in week 4 of the 12-week intervention. Weekly PA goals were incrementally increased by 10% of the participant’s previous week’s average steps per day. For example, a participant achieving 5000 steps in week 1 would receive a goal of 5500 for week 2. Youth meeting the 12,000-step recommendation for adolescents were encouraged to maintain this goal. Similarly, weekly sleep goals increased successively by 20 minutes of the previous week’s average hours of sleep per night. Youth who achieved sleep recommendations of at least 7 hours of sleep per night were encouraged to maintain this sleep goal. Youth randomized to the waitlist control group received a Fitbit Charge 5 device and a one-page informational handout on PA and sleep guidelines. After completion of the 12-week intervention period, youth in the waitlist control group received the daily SMS text messages grounded in SDT for an additional 12 weeks.

A convenience sample of youth originally randomized to the intervention (N=15) were invited to participate in an exit interview to evaluate intervention feasibility and acceptability following the 12-week intervention period. A semistructured interview guide consisting of 12 open-ended, nonleading questions was developed by the investigative team to elicit information on perceptions of the SMS text messages, wearing and using the Fitbit, the design and implementation of the intervention, and suggestions for improving the intervention. During the co-design process, there was interest among some youth in having peers provide social support throughout the intervention. While the final intervention remained individually focused, the exit interview provided the opportunity to ask participants about unprompted, organically received peer social support during the intervention, their perspectives on the importance of peer social support for behavior change, and suggestions and preferences for peer inclusion in DHIs like Fit24. Interviews were conducted by research assistants trained in qualitative methods. Probes, prompts, and clarification were used to expand, explore, and understand responses. Two interviewers conducted all interviews in-person in a private room or by telephone using a dedicated study phone line. All interviews were digitally recorded, and participants were compensated US $25 for their time and participation.

### Statistical Analyses

Interviews were professionally transcribed verbatim, reviewed for accuracy, and coded by 2 coders using the qualitative analysis software program NVivo (version 12.5, QSR International). An a priori codebook, containing codes and definitions, focused on the participant’s perceptions of (1) the SMS text messages, (2) wearing and using the Fitbit, (3) the design and implementation of the intervention, (4) preferences for peer social support, (5) suggestions for integrating peer social support into DHIs, and (6) overall suggestions for improvement. Following this, coders independently read each transcript within the software program and applied codes based on the goals of the interviews. Once the first reading was completed, coders met to compare codes used within each transcript. At this time, codes were either confirmed, modified, or rejected, and the codebook (codes and definitions) was updated accordingly. Using the updated codebook, coders followed the same process of independently applying codes to interview transcripts to complete 3 additional independent readings of the transcripts [15]. Coding was discontinued when it was determined that theoretical saturation in relation to study objectives was attained [16].

The Practical, Robust Implementation, and Sustainability Model (PRISM) provides a framework for translating feasible and efficacious interventions into practice settings [17,18]. Using a structured coding analysis approach, codes from the codebook were categorized into dimensions of the PRISM framework. Codes within each dimension were then grouped together to identify themes within each dimension. In this analysis, themes represent repeated ideas or common perceptions among the youth. Because this study is in the pilot stage, only the participant perspective dimensions of the PRISM were used, as these dimensions evaluate feasibility and acceptability. The operational definitions for participant perspective dimensions are presented in Table 1.

**Table 1:** Operational definitions of Practical, Robust Implementation, and Sustainability Model (PRISM) dimensions.

Prism dimension				Operational definitions
**Acceptability**				
		Participant centeredness		Whether the program addressed the values, preferences, and needs of the target population.
		Participant choices		How the participant chose to use the Fitbit device and chose to respond to SMS text messages.
		Services and access		Perspectives on the services provided by the research team and accessibility of the intervention.
		Feedback of results		Perspectives on participant’s own progress through the program, suggestions for improvement, and suggestions for integrating peer social support.
**Feasibility**				
	Participant barriers		Challenges and barriers to participating in the intervention and to meeting activity and sleep goals.	
	Seamlessness between program elements		Technical issues experienced in using and integrating the SMS text messaging service and Fitbit device.	
	Burdens (complexity and cost)		Perspectives on program elements that may have caused burden (eg, financial cost and confusion).	

### Ethical Considerations

This research received approval from the Institutional Review Board of the Baylor College of Medicine (H-49195) and was reviewed on an annual basis. Parental consent and youth assent for participation in this feasibility pilot study and follow-up exit interview were obtained before any study procedures. Youth were compensated US $25 for participation in the exit interview and were compensated separately for their participation in the 12-week intervention and completion of pre- and postintervention assessments (US $200). All data obtained through this study were deidentified and stored on a secure network drive at Baylor College of Medicine that is specifically designated for the storage of sensitive and confidential data. All data files were password-protected. Data access was restricted to those with appropriate institutional review board authorization and limited to principals, coinvestigators, and statisticians.

## Results

### Overview

Interviews (N=15) were conducted between January and March 2023 and lasted about an average of 33 minutes. All participants chose to conduct the interview in English. Baseline descriptive and anthropometric data are presented in Table 2.

**Table 2:** Participant characteristics (N=15).

Variable			Value
**Gender, n**			
	Male		7
	Female		8
Average age (years), mean (SD)			14.87 (0.91)
Average BMI (kg/m^2^), mean (SD)			33.6 (7.5)
**Parent-reported annual household income (US $), n (%)**			
		10,000-19,999	2/15 (13.3)
		20,000-29,999	3/15 (20)
		30,000-39,999	5/15 (33)
		>40,000	3/15 (20)

The themes within each PRISM dimension are presented in Textbox 1 and are supported by exemplary quotes. Themes could fit within multiple PRISM dimensions, based on a priori definitions. To demonstrate the diversity of responses, quotes are presented from both male and female participants. Using Table 1 as a guide, the PRISM was used to make a determination of intervention acceptability and feasibility.

**Exemplary quotes categorized by the Practical, Robust Implementation, and Sustainability Model (PRISM) dimension.**

**Themes and exemplary quotes**

- Participant centeredness
  Tracking health data:
    “...one of my favorite parts about the program was the Fitbit watch. I will use it to track whenever I would go on runs. Check my sleep, I would constantly check my sleep. And that helped me stay more motivated...” (female adolescent)
    “I used to check my stats and my heartbeat, because I like to look at that…” (male adolescent)
  Texts were motivating and encouraging:
    “...just knowing that there’s somebody out there who knew I could do it, and it was somebody out there telling me that I could do it.” (male adolescent)
    “I love the text messages and loved how y’all, umm, sent motivational texts and told me like how I could do it...” (female adolescent)
  Goals were motivating:
    “It was just having this step goal like it would motivate me to walk more to complete that goal and to sleep. Like, I know like what time I should go to bed and what time I should wake up...” (female adolescent)
    “I guess you could say motivational...Like, I feel like I’d take it a little as a competition and be like if I finished them (the goals), like I actually accomplished my goal for the day.” (male adolescent)
  Texts support youth’s needs:
    “They’re (text messages) motivating me to keep going. The fact that it told me, here’s some good workout decisions and ways to get better to get more steps and things just to help me get better.” (male adolescent)
    “They (text messages) were very informative...It was like, it’s how much you’ve done this week, this is how much we want you to do next time, you know, these are some things you can do. Just to check in and it was like, very engaging.” (female adolescent)
- Participant choices
  Track health behaviors:
    “So, whenever I would go on walks, I would go on runs, I would start the running time or the fitness part of the watch. It would track like how much I ran and what pace...” (female adolescent) “I use it to help me stay active and you know, just track my calories like I said before, and just help me stay active and like motivate myself to do more and do better.” (male adolescent)
  Friend support:
    “They (friends) would encourage me, they would encourage me to do more steps and be more active in school.” (male adolescent)
    “They (friends) asked me to workout with them or go on walks with them.” (female adolescent)
- Service access
  Timing of SMS text messages:
    “...the messages, like how I said they should probably be sent in a later time just because of school.” (female adolescent)
- Feedback and results
  Need for personal responsibility:
    “I would like some support, of course, but at the end of the day, it’s on me. I’m the one responsible for keeping up my goals and motivating myself to do it.” (female adolescent)
    “I think it’s very important to have friends that motivate you to do better...but also it takes some will from yourself to keep yourself motivated to continue to be healthy.” (female adolescent)
  Increased activity and sleep:
    “I wasn’t that active but the more I got into the program, I got more active, and I started doing more stuff around my house and things like that.” (male adolescent)
  Peer support desired:
    “I like having friends cheer me on when I do things.” (female adolescent)
    “I like not doing this alone. Like, if I’m struggling...it’s just nice knowing that you’re not doing this alone.” (male adolescent)
  Improve timing of SMS text messages:
    “...maybe sending messages at a later time after school hours would probably be something that I would recommend.” (female adolescent)
    “What I like least about the program was the time because sometimes I would get on my watch, like, an alert to start moving, but I couldn’t because I was at school.” (male adolescent)
- Participant barriers
  Fitbit watch band: “...there was a time when I wore the watch with the original strands. Boy, that irritated my skin a lot. And it was a bit on the uncomfortable side.” (male adolescent)
  Environmental barriers:
    “...we don’t live in a good neighborhood, so most of my walking would have to be inside, in the backyard, which is like muddy...you can’t do much around here.” (female adolescent)
    “...like there was a period where there was a lot of schoolwork...I didn’t really have much time to like, do physical activities...” (male adolescent)
- Seamlessness between program elements
  Sync issues:
    “Sometimes the steps were wrong, but that was a quick fix.” (female adolescent)
    “My Fitbit wouldn’t sync, or like, it would keep logging me out. And I would have to constantly restart the app.” (female adolescent)
- Burdens: no burdens discussed

### Intervention Acceptability

#### Participant Centeredness

Most participants had positive perceptions of wearing the Fitbit device, and almost all participants had positive perceptions of SMS text messages, indicating that the use of these digital tools was acceptable among this population. Participants valued wearing the Fitbit and the ability to track activity, sleep, and other health information (eg, calories and heartbeat). Participants found the messages to be motivational and encouraging, reporting that the SMS text messages increased their self-efficacy to be physically active, meaning their belief that they could engage in PA. The youth also reported that the step and sleep goals were motivating, with some sharing that they felt encouraged to try harder and push themselves to achieve their goals. Youth shared that the SMS text messages were supportive as they provided new information regarding health behaviors, activity suggestions, and encouraging feedback on goal progress and attainment.

#### Participant Choices

Participants used the Fitbit device in several ways, including using it to track their PA, sleep, and diet behaviors. They reported that it was motivating to be able to track their progress and behavioral achievements. While diet was not a component of the intervention, many participants reported that they used the Fitbit app on their smartphones to keep track of their caloric intake and expenditure. While this was designed as an individual intervention, almost all participants reported that they organically received friend support while participating in the intervention. For many youth, this was reported as instrumental support in the form of friends engaging in activity with the participant and emotional support in the form of encouragement and motivation.

#### Service and Access

While the content of the SMS text messages was found to be acceptable, many youths shared that the timing of the messages made it difficult to follow through on activity suggestions. Messages were scheduled to be sent at 8 AM in the morning and 4 PM in the afternoon. Participants expressed frustration when texts were received in the afternoon as most participants were still at school, which often meant they could not follow through with a behavior prompt or access their phone to read or respond to messages.

#### Feedback on Results

Youth reported several perceived benefits from participating in the program, such as increased sleep and PA. Participants also discussed feeling increased motivation for PA and sleep and increased PA self-efficacy. Although many participants organically received peer social support during the intervention, when asked about the importance of peer support, most stated that peer support was somewhat to not at all important. The most recommended suggestions for how to include peers in the Fit24 intervention included working as a team with peers to achieve a common goal or complete a challenge and a group chat where youth can receive peer support for goal attainment. While this peer component was desired by some youth, many expressed the need to recognize the importance of personal responsibility for behavior change. This was discussed as the need to take responsibility and develop one’s “self-will” for improving PA and sleep. Lastly, in line with issues with the timing of texts, youth recommended sending SMS text messages later in the day to ensure that they are home and able to act on the behavioral prompts that are sent.

### Intervention Feasibility

#### Participant Barriers

One of the barriers related to participation included issues with the Fitbit device watch band. The band is made of an elastomer material, and several participants reported the material was irritating to the skin. Additionally, some participants experienced environmental barriers that limited their ability to act on activity suggestions, including a lack of access to PA resources, responsibilities at home (eg, chores and family obligations), and school-related barriers such as spending too much time at school or having too much homework. These barriers represent areas for improvement to increase the acceptability and feasibility of wearing the device and engage youth in suggested PA activities.

#### Seamlessness of Transition Between Program Elements

Some participants reported sync issues, which did not allow the research team to seamlessly integrate Fitbit data into the SMS text messages. To integrate Fitbit data into the SMS text messages, we used Fitabase, a data collection and analysis platform for internet-connected devices. Our messaging platform, Mosio, used the Fitabase app programming interface to provide participants with personalized guidance on goal setting and feedback on goal attainment using data extracted from each individual Fitbit. However, due to sync issues, the most accurate and up-to-date data were not always available within Fitabase, causing some participants to receive SMS text messages with inaccurate feedback on goal attainment.

#### Burdens

One participant mentioned the burden of having to charge the Fitbit device; however, this was only mentioned by one adolescent and therefore did not emerge as a theme. There were no other burdens on participation reported by any participants.

## Discussion

### Overview

The purpose of this study was to qualitatively evaluate the perspectives of Hispanic adolescents at risk for diabetes on the feasibility and acceptability of the 12-week Fit24 intervention. Using the PRISM as a guide, the Fit24 intervention was deemed to be both feasible and acceptable based on participant feedback. These findings are important, as few studies have developed DHIs for Hispanic youth, and even fewer have engaged youth in the development and evaluation of DHIs [9,13]. As the integration of technology into health promotion and disease prevention strategies continues to increase, it is important to identify feasible and acceptable intervention strategies for engaging youth, especially high-risk youth like Hispanic adolescents with obesity who are disproportionately impacted by diabetes [19].

Many participants valued and chose to engage in tracking their health data, including steps, sleep, and caloric intake, a behavior that was not promoted in the intervention. According to self-regulation theory, tracking or self-monitoring leads to behavior change as it encourages self-evaluation of progress toward a behavioral goal and positively reinforces the behaviors that lead to the achievement of a behavioral goal [20,21]. Self-monitoring is an automated feature in the design of devices like personal activity trackers [22]. Previous research has highlighted the need for more DHIs to leverage this function [23] and suggests that the increased use of self-monitoring may lead to increased engagement with the device [24]. Given the high level of acceptability of this feature, our findings also support the increased use of self-tracking in DHIs. Youth reported that the goal-setting, and SMS text messages received throughout the intervention were motivational, encouraging, and supportive of their needs. In the co-design process used to develop the bank of SMS text messages, youth expressed a need for messages that were encouraging and used motivating words to support behavior change [13]. The youth also informed the research team how to provide assistance in goal setting in a manner that preserved their autonomy, a key construct within the SDT. In doing so, the research team recommended step and sleep goals that youth could accept, or they could set a different goal [13]. Findings from this study validate the importance of collaborating with youth to develop messaging content and implement behavior change techniques that are acceptable among the population of focus [9].

Peer social support can shape the development of health behaviors and has been shown to lead to increased PA among Hispanic adolescents, including those with obesity [25,26]. Based on participant responses, the organic peer support received throughout the intervention included not only friends but also family such as parents, siblings, and cousins. If a peer component were to be added to Fit24, youth would express a desire for emotional and instrumental support through group chats or team challenges. This is consistent with previous literature that has reported emotional and instrumental support as the most common type of support received by youth in this population [27]. However, there are currently no peer supported DHIs focused on diabetes prevention among adolescents. Given that such a high level of support was received, it was surprising that participants placed such a low level of importance on receiving peer support for behavior change; instead, youth emphasized the need to take personal responsibility for changing their health behaviors. This finding suggests that while peer social support is welcomed, it should be used as a tool to foster and promote personal ownership of behavior change so that the desired behavior becomes internally and autonomously motivated. This is in line with other studies that have also reported that adolescents desire support to foster internal motivation for behavior change [28]. Future studies should continue to increase our understanding of peer social support among Hispanic adolescents and how to integrate this key behavioral determinant in future DHIs like Fit24.

There were several limitations to this study, including issues related to the feasibility of the Fitbit component of the study. To start, this was a short-term intervention, and previous research has shown that Fitbit use and self-monitoring decline after 12 weeks. Longer intervention periods with long-term follow-up are needed to more comprehensively understand the feasibility and acceptability of sustained use of trackers and their ability to sustain behavior change among this population [29]. Additionally, several participants reported that the Fitbit watch band was uncomfortable, with several participants reporting that the band irritated their skin, an issue that has been reported by others [30]. This issue was remedied by purchasing hypoallergenic fabric bands. Additionally, participants discussed sync issues, which at times caused frustration and led to feedback messages that inaccurately reported progress toward behavior goals. This issue has also been previously reported in several studies conducted among adults [30-32]. The Fitbit device syncs through the Fitbit app once a Bluetooth connection has been established, allowing the participant and the research team to remotely monitor the data. Often, the app is set to “sync automatically,” meaning it is running continuously in the background, allowing for real-time monitoring of data. However, sync issues occurred in this study due to a loss of Bluetooth connection, differences in app settings, or differences in operating systems that did not allow for the automatic or continuous syncing of the device. Using guidance from previous studies, data were closely monitored to the best of our ability; participants received a weekly reminder to sync their devices, and an individual SMS text message if no data were visible after 2 days [31-33]. Future studies should troubleshoot activity trackers across different types of smartphone devices, operating systems, and settings. Additionally, investigators should have a contingency plan for recovering data should sync issues occur to minimize data loss and feelings of frustration among participants. Participants did not discuss any burdens associated with the Fitbit, SMS text messages, or participation in the intervention, which adds to the acceptability and feasibility of this program.

The youth from this study made several recommendations for improving the intervention. Many students reported that they were still at school when afternoon messages were received. This limited the participant’s ability to engage with the messages given the limitations within the school environment and school rules regarding phone use. Future studies should consider personalizing the timing of SMS text messages given the diversity in school start and end times and different youth schedules in general. Participants also highlighted the need to be more considerate of environmental barriers that limit opportunities for engaging in PA. Activity suggestions made within SMS text messages should be revised so that, when possible, outside resources are not needed, and alternatives should be provided for overcoming potential environmental barriers.

### Conclusion

The use of a Fitbit and SMS text messaging to promote PA and sleep among Hispanic adolescents with obesity was acceptable and feasible; however, several issues were identified that need to be addressed before the next iteration of this study. The behavior goals and daily SMS text messages in this study were perceived as motivating and encouraging for goal achievement. Youth were highly engaged in self-monitoring their behaviors, and future work should examine self-monitoring as a key behavioral driver in DHIs. Our findings were not clear regarding the integration of peer social support within DHIs like Fit24. Youth feedback suggests that peers should be broadly defined to include family and friends and should be used in a manner that is considerate of the youth’s need to establish internal motivation for behavior change. Considering this feedback, feasible and sustainable strategies for fostering internal motivation should be explored in future research. Solutions are needed to address sync issues with the Fitbit device, and the timing of when SMS text messages are sent should be personalized to the participant’s schedule for greater engagement around behavior prompts. Findings from this study can inform the design and implementation strategies of future DHIs among high-risk pediatric populations. Future trials are needed to test the efficacy, reach, and sustainability of Fit24 among Hispanic adolescents at risk for diabetes.

## Abbreviations

DHI: digital health intervention
PA: physical activity
PRISM: Practical, Robust Implementation and Sustainability Model
SDT: self-determination theory

## Data Availability

The data sets generated and analyzed during this study are not publicly available because of institutional ethics guidelines and the constituents of the approved ethics application. The data generated may be made available to the first author (EGS) conditional upon approval from our institution’s ethics board.
